# Kaposi’s Sarcoma: A Non-Communicable Outcome Mainly Prompted by Communicable Diseases in Sub-Saharan Africa

**DOI:** 10.3390/ijms262010198

**Published:** 2025-10-20

**Authors:** Anthony Idam Mamimandjiami, Jéordy-Dimitri Engone-Ondo, Pamela Moussavou-Boundzanga, Augustin Mouinga-Ondeme, Ivan S. Mfouo-Tynga

**Affiliations:** 1 Department of Biology, Faculty of Sciences, University of Sciences and Technology of Masuku, Franceville P.O. Box 943, Gabon; 2 Unit of Retroviral Infections and Associated Pathologies, Department of Virology, International Centre for Medical Research of Franceville, Franceville P.O. Box 769, Gabon

**Keywords:** Kaposi’s sarcoma-associated herpesvirus (KSHV), co-infections, KS-pathogenesis, Sub-Saharan Africa

## Abstract

Kaposi’s sarcoma (KS) is a tumor that primarily affects the skin, caused by a multifactorial pathogenesis mediated through immune dysfunction, often leading to increased morbidity and mortality in Sub-Saharan Africa (SSA). Human herpesvirus-8, also known as Kaposi’s sarcoma-associated herpesvirus (KSHV), induces an infection that can facilitate the pathogenesis of KS and other conditions. All KSHV subtypes depend on the expression of specific markers, such as K1 proteins, which play critical roles in their life cycles. The infection is unevenly scattered worldwide, with individuals infected with human immunodeficiency virus (HIV) and pregnant women being among the most vulnerable groups. HIV infection and related effectors, such as TAT proteins, have substantial impacts on KSHV infectiousness, angiogenesis, various signaling pathways, and KS pathogenesis. Africa endures the heaviest burden of KS, which affects both men and women, sometimes from an early age. KS’s pathogenesis and underlying mechanisms remain unclear; this study aims to highlight the dynamics to be considered in managing and mitigating the burden of KS in SSA. In that region, certain infections are endemic and can cause intermediate health damage leading to KS tumorigenesis, highlighting the link between non-communicable and communicable diseases.

## 1. Introduction

Kaposi’s sarcoma (KS) is a skin tumor characterized by angioproliferation arising from infected endothelial or progenitor cells [1]. The etiological agent of all types of KS is the human gamma-herpesvirus-8 (HHV-8), also known as Kaposi’s sarcoma-associated herpesvirus (KSHV), which was first isolated in 1994 from a skin sample of a patient who had conditions both related and unrelated to human immunodeficiency virus/acquired immunodeficiency syndrome (HIV/AIDS) [2]. Usually, this herpesvirus is isolated from samples of patients with AIDS, who have a higher risk of developing KS [3,4]. It has been observed that KS malignancy strongly mirrors the HIV epidemic, and the majority of KS occurrences are identified in KSHV-infected patients, who also suffer from immune dysfunction. Thus, immune dysfunction provides suitable conditions for KS development [5,6]. The KSHV agent is consistently present in certain regions of the world, including Sub-Saharan Africa (SSA), the Mediterranean Basin, the northwestern part of China, and South America, particularly among Amerindian populations [7,8,9,10]. The levels of KSHV infection are predominantly high in Western subpopulations of men having sex with men (MSM). Additionally, HIV infection seems to be more prevalent among MSM in comparison to the heterosexual population. Consequently, KS is more common in MSM with AIDS than in other subgroups of adults with AIDS [11,12]. The incidence of KS is higher in KSHV-endemic populations, and the virus also causes other conditions, including primary effusion lymphoma, multicentric Castleman disease, and many related lymphomas [13,14,15,16]. Clearly, there exists no dichotomy, but rather a multitude of nuances, as a synergy between non-communicable and communicable diseases often occurs, involving infectious agents and other factors that impair immune and homeostatic functioning.

According to the Joint United Nations Programme on HIV/AIDS (UNAIDS), in 2023, 1.3 million people were newly infected with HIV. This infection accounts for more than 42.3 million lives lost globally since the beginning of this epidemic, and more than 630,000 deaths in 2023 [17]. Although there is still no conclusive cure, HIV infection can be controlled with appropriate treatment and continued care. Globally, an estimated 39.4 million people living with HIV (PLHIV) were reported in 2023, and more than two-thirds of PLHIV are on the African continent [17,18]. Many PLHIV are also suffering from a large number of pathologies, leading to immune disorders and further infections [18,19]. HIV infection is often characterized by weaker immune responses that facilitate other conditions; viral co-infections are commonly identified in patients with HIV, especially in those infected with viruses that share common routes with HIV [20,21]. While certain infections are silent when co-infecting and do not affect the course of HIV, and vice versa, others impact both the history of HIV infection and outcome of treatments [22]. Some agents and/or pro-oncogenic viruses induce cancers and pathologies more readily when co-infecting patients with HIV than other individuals in the general population [13,23]. Due to synergistic actions, various co-infectious agents cause more deleterious effects than when they are in a mono-infectious condition, and antagonistic effects are seldom observed in most cases. Therefore, diagnosis of certain pathogens or potential co-agents (opportunistic agents) should be considered before designing a treatment regimen for patients with HIV [24]. Persistent inflammatory responses and non-AIDS-related conditions are often observed in patients infected with HIV; this could be due to the presence of KSHV and/or other related pathologies in patients infected with HIV; thus, a co-infection (HIV-KSHV) in a specific population (HIV group) and/or region is possible [25]. The co-pathogen KSHV has been associated with increased inflammation and activated immunity in HIV-infected individuals. Therefore, it is well established that certain practices, such as sexual behaviors, might influence or play roles in HIV transmission and infection, which in turn are associated with increased risks of KSHV seroprevalence. KSHV infection may not necessarily be related to sexual behavior in African regions, where a high prevalence of KSHV-HIV co-infection has been reported [26,27].

Although KSHV stands out as the main etiological agent for KS, the development of this tumor is influenced by a variety of additional cofactors, which can further impair immune function. In this study, the crosstalk between the infectious agent (KSHV) and a key risk factor (HIV) for KS is thoroughly discussed, as this co-infection weakens immune function and appears to be a significant contributor to KS. The evidence of co-infection and a few other factors present in SSA regions is highlighted before KS-induced mechanisms are analyzed, together with possible eradication measures.

## 2. Crosstalk Between HIV-KSHV Co-Infection in SSA

### 2.1. Epidemiology and Risk Factors of KSHV in Africa

Many diseases have been reported in patients infected with HIV and/or immunosuppressed individuals following KSHV infection. The low prevalence of KSHV-associated diseases in individuals without HIV highlights the major role of the co-infection HIV-KSHV in the onset of diseases [28,29]. Co-infected individuals may develop comorbidity, which is the simultaneous presence of two or more diseases at a given time. Immunosuppressed individuals may have a higher risk of developing KSHV-related conditions, as these conditions tend to be asymptomatic or non-existent in the absence of an immune deficiency [30,31]. As previously indicated, the prevalence of KSHV infection tends to be elevated in MSM groups than in the general population. The infection is even higher in MSM subgroups infected with HIV in the USA, where the prevalence of KSHV infection in that particular subgroup was reported to be twice that of the counterpart population of men without HIV [32]. Due to cultural and ancestral influences, the MSM population in SSA is relatively small when compared to the Western parts of the world. However, the prevalence of KSHV infection is surprisingly high in SSA [33,34]. Additionally, the transplantation of organs, which causes immunosuppression, is not commonly practiced in SSA due to the cost and lack of medical facilities to perform such dedicated operations. Thus, factors other than sexual orientation may have intervened and facilitated the high prevalence of KSHV infection observed in SSA [35,36]. Saliva is commonly used among SSA-inhabitants and MSM, and this could explain the high KSHV prevalence observed in these groups. Saliva could be considered an important factor involved in certain feeding habits, as it is used in food pre-mastication for children and/or common dishes for adults in SSA, elucidating the fact that KSHV infection was reported to be high in children and even more prevalent in rural areas, as observed in Uganda [37,38]. Also, saliva could be considered a lubricant in anal/oral sex in MSM groups [27].

**Figure 1:**
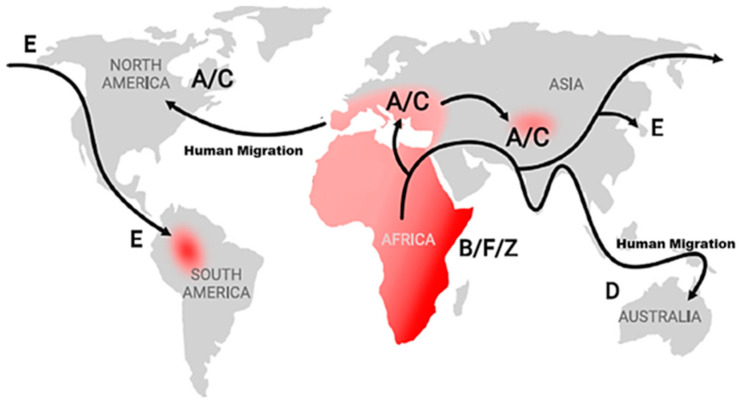
Global distribution of KSHV subtypes. The evolution and dynamics of KSHV infection can also reflect human migration and history (adapted from Ref. [44]).

Many subtypes of infection have been identified by analyzing KSHV samples from around the world. Among them, five major subtypes have been recognized, with A and C subtypes being closely related and often considered together [39,40]. The A/C subtype was predominantly identified in two distinct regions, accounting for 5% of those in Western countries (the USA, Northern Europe, and South Asia) and 10–20% in Mediterranean countries (Italy, Greece, and North Africa) [40]. The B subtype was mostly localized in SSA, accounting for the majority (more than 50%) of all patients infected with KSHV. Those from the Amerindian regions and the Pacific islands, including Japan, were found to have the E and D subtypes, respectively [40,41,42]. KSHV infection is not homogeneously spread throughout; an atypical F-subtype has been described, but only among Ugandan citizens [43]. The subtypes enable the mapping of the infection and differentiation between various epidemiological regions worldwide. Additionally, they help determine pathogenicity, types of associated pathologies, potential mutations (such as resistance), and the onset of initial symptoms or the manifestation of associated pathologies [34]. Finally, the global distribution of KSHV subtypes and infection can depict human migration and history (Figure 1) [44].

**Figure 2:**
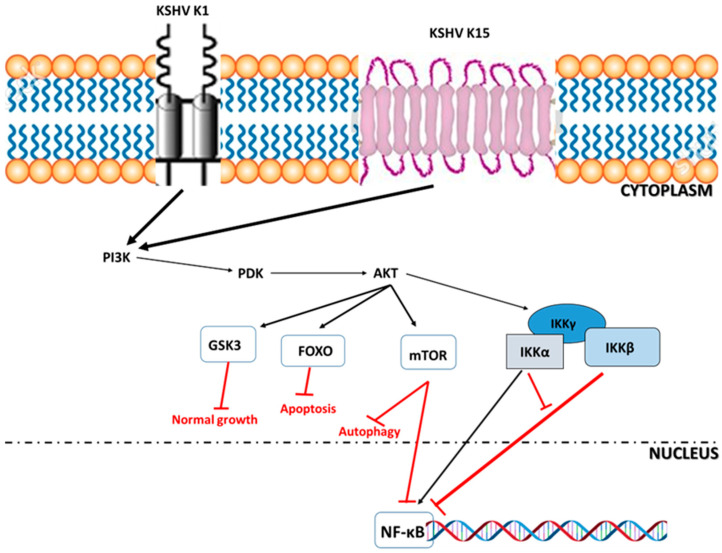
Activation of the KSHV lytic phase through the PI3/AKT/mTOR signaling pathway. Both KSHV K-1 and -15 proteins can trigger the cellular activation of the phosphatidylinositol 3-kinase (PI3K)/Akt/mTOR pathway, leading to the phosphorylation of downstream effectors. The glycogen synthetase kinase-3-beta (GSK3) associated signaling is crucial to virus infection of cells. The members of class O forkhead box transcription factors (FOXOs) have important roles in metabolism, including cellular proliferation, stress resistance, and apoptosis. The mammalian target of rapamycin (mTOR) is a protein kinase that regulates cellular metabolism, catabolism, immune responses, autophagy, survival, proliferation, and migration, thereby maintaining cellular homeostasis. Furthermore, the lytic replication and growth of KSHV-infected cells are promoted by levels of effectors (IKK mediators) of the transcription nuclear factor (NF-κB). The subunit IKKα can both suppress IKKβ and activate NF-κB. The nuclear factor controls the maintenance of viral latency and represses lytic replication.

Genomic variability mainly defines the division into subtypes, and three genes at open reading frames (ORFs) -26, -75, and -K1 have critical importance in distinguishing the subtypes [45]. The first gene (ORF-K1) of the viral genome encodes for a protein that can modulate signaling pathways and cell survival [46]. The gene contains two important regions (VR-1 and VR-2), and the specific features of the K1 gene characterize all the subtypes. This particular portion of the genome encodes a signaling transmembrane protein involved in the lytic phase, when it is highly upregulated but downregulated during latency [46]. This signaling K1-protein has critical functions in the KSHV lifecycle, including the activation of the phosphatidylinositol 3-kinase (PI3K)/Akt/mTOR pathway, cell transformation, and cell death, as illustrated in Figure 2. The upregulated signaling pathway mediates lytic replication, viral gene expression, and subsequent cell survival in various cell types, including B and endothelial cells [47,48]. The K1-expressing cells facilitate angiogenesis by activating the vascular endothelial growth factor (VEGF) and subsequently vascularization [49]. Despite a lack of data in some countries, the K (-1 and -15) genes are the most commonly occurring genotypes, present in approximately 60% of available evidence. The K-1 genotypes have been described in almost 40% of work carried out in Africa [46,48,50]. Interestingly, high prevalence rates were reported in all regions of Africa, indicating a generalized KSHV infection and the endemic status it has received.

### 2.2. Description of the Prevalence of KSHV Infection in SSA Countries

The seroprevalence rate designates the proportion of a population that tests positive for specific markers of a viral infection as measured in blood serum. The development of complex forms of KSHV-associated diseases often occurs in SSA, especially when the seroprevalence rates of KSHV infection are high or KSHV-HIV co-infection is reported. In comparison to European countries, the rates of detectable anti-latent and anti-lytic markers in SSA are slightly elevated and up to six times higher, respectively, than those in these markers [37,51,52,53].

Reports have revealed that the epidemiologic features of KSHV infection differ according to the populations studied and their HIV status, as well as other factors. Prior to the 1980s and the discovery of HIV, symptomatic signs related to KSHV-associated diseases had already been reported in a dozen SSA countries. More than four decades later, KSHV-related diseases as well as KSHV prevalence rates are reported to be high in most parts of SSA countries [26,37,54]. Certain countries have high prevalence rates of KSHV, with the national average and the highest rates exceeding 50% and 85%, respectively [53,55,56]. The epidemiologic patterns of KSHV infection are unevenly scattered within African regions. The seroprevalence rates remain elevated in central and eastern parts of Africa, and relatively low in the western and northern regions. These main SSA regions have been identified, and their subpopulation groups exhibit specific features of the infection. In Senegal and around the region of Dakar (capital city), a KSHV seroprevalence rate of 14.3% was reported in pregnant women. In comparison, a slightly lower rate of 12.7% was observed in the corresponding group in Ouagadougou, which was similar to the rate of KSHV infection in blood donors in Burkina Faso [57,58]. The group infected with HIV in Nigeria exhibited a seroprevalence rate of 62%, which was almost 2.5-fold higher than the estimated 26% seroprevalence rate in the group without HIV [59]. A 100% KSHV seroprevalence rate was observed in a group of Ghanaian KS patients; the obtained rate was more than double that in the general population, and these figures did not differ much over time [60,61]. As the etiologic agent of KS, KSHV is identified in almost all KS patients, although not all KSHV-positive individuals will develop KS or associated diseases. Substantial efforts had been made in the South-Eastern region of SSA to understand and control KSHV infection. In the Ugandan rural population, the KSHV infection was reported to be prevalent, with an estimated prevalence of 49% in the adult group, and the infection also remained elevated in children [38]. The seroprevalence rate was estimated to be 40–50% in the general population of Uganda; however, a higher rate was reported in Botswana, ranging from 55 to 90% [62,63]. During the same period, a lower rate of 21.4% was observed in Mozambique. After analyzing various socio-demographic factors, some appeared to be determinants, having been linked to increasing or higher (>50%) seroprevalence rates of KSHV infection [62,63,64,65].

In Tanzania, KSHV infection was commonly diagnosed in female citizens, particularly in rural areas. The rural town of Tosamaganga had a seroprevalence rate of 46.3%, which was three times higher than that of the densely populated and touristy area of Pemba Island (14.4%) [65,66]. According to a study conducted in Ethiopia, pregnant women had a seroprevalence rate of 50%, approximately 10% higher than that of the group of women infected with HIV; much lower rates were observed in women without [67]. A seroprevalence rate of 46% was reported in patients with HIV, who were asymptomatic, both in Uganda and Zambia [58]. In the two countries, a seroprevalence rate of 50% (anti-latent/anti-lytic markers) was reported in children suffering from sickle cell anemia [68]. In another study performed in Kenya, the same markers were reported at a rate of 43% and showed an increase of up to 68% when evaluated in the capital city [69,70]. The young adult group in Zimbabwe had a relatively low seroprevalence rate of those markers, estimated at 13%. In comparison, the endemic Malawi showed an important prevalence from childhood that kept increasing to 54% in the general population and up to 90% in groups with HIV [39,71,72]. In South Africa, the KSHV markers were reported at a relatively low rate of 30% in the general population in some regions, but increased (48%) in groups with HIV [73].

The central African region is one of the most endemic areas in the world for KSHV infection and associated diseases. Additionally, more KS cases have been reported in children when compared to other regions, and most are non-related to HIV infection, with the peak being around age 5. The KS belt, located across the equatorial line, has been described as indicating a higher prevalence and greater variation associated with KSHV infection. The seroprevalence rate was elevated from childhood, and by the age of puberty, the figures were similar to those observed in many adult and/or high-risk groups from other SSA regions [74,75]. In Cameroon, a seroprevalence rate of 50.5% was observed among blood donors, with similar serologic markers of anti-latent (50.5%) and anti-lytic (25.3%) in the northern region. However, pregnant women exhibited national rates of 27.5 and 60%, respectively, for those markers [76,77]. A strong correlation was found between mothers and their children after analyzing samples from more than 600 individuals in 92 families. A 60% rate for anti-latent markers was also detected, along with 30 and 62% KSHV prevalence rates in children under 9 and 15 years old, respectively [76]. An infection rate of 37% was reported in 2063 individuals living in a southern and rural region near the border of Gabon [78].

An analysis of data obtained from Bantu and Pygmy populations in Southern and Eastern Cameroon demonstrates a high seroprevalence of KSHV within these two major groups. The circulating strains are characterized by the presence of both the highly genetically diverse A5 and the B K1 subtypes [79]. One study evaluating the level of KSHV infection in pregnant women in Lambarene, a city at the center of Gabon, showed a 35% seroprevalence rate [72,76,80]. Studies in the Central African Republic (CAR) reported an 82% prevalence rate and listed CAR as one of the most endemic regions for KS, KSHV, and HIV in the world [81,82]. Numerous cases of KSHV-associated disorders in those countries were also reported, attesting to the presence of alarming infections. Table 1 summarizes the estimated KSHV seroprevalence and KS incidence across SSA countries, indicating that the K1 (A5 and B) and K15 (P and M) are among the most commonly found KSHV subtypes, with the infection being unevenly scattered, and affected by several cofactors, including the studied populations, living conditions, and areas (urban or rural), and health status. The infection rate is relatively high in central, eastern, and southern Africa, while elevated KS incidences are observed in countries like Cameroon, South Africa, Zimbabwe, and Zambia.

### 2.3. Transmission and Pathogenesis of KSHV

The modes of transmission of KSHV infection appear to differ between low- and high-endemic areas. In low-endemic areas, the infection is mainly present in the MSM population, where the virus is thought to be transmitted during sexual contact. The anal route may constitute a vulnerable entry point during sexual intercourse, as the rate of KSHV transmission remains elevated in MSM even when so-called safe-sex practices are employed [95]. Saliva is often used as a lubricant by MSM during deep oral kissing, anal, and anal–oral intercourse. The transmission seems not to be associated with sexual behaviors in this case, nor with HIV status, as MSM without HIV still showed a higher prevalence of KSHV infection and associated pathologies [96,97,98]. In contrast, in high-endemic areas, such as in central African countries, KSHV transmission occurs mainly from mother to child and between siblings. Saliva seems to play a major role in viral transmission, serving as a reservoir for KSHV [99]. Furthermore, the presence and analysis of the viral DNA in saliva reveal high viral loads, suggesting that saliva is the main fluid responsible for transmission. Therefore, the oral route seems to be a critical point of entry and transmission in SSA, where the infection can occur and be facilitated during childhood by certain cultural behaviors such as food pre-mastication and/or close contacts [100,101,102]. The transmission of KSHV might happen at an early age so that by puberty, certain adolescent groups have already shown an infection rate comparable to that seen in adult groups. The viral load of KSHV is significantly low in semen, thus implying that sexual intercourse could not be a major means of transmission [101,102]. Therefore, heterosexual transmission remains low, as well as transmission through blood products. Thus, blood transfusion may be rarely considered as a means of spreading the infection; it is associated with a relatively low risk and rate, even in people living in areas where KSHV prevalence is high [103].

A few genes can control the latent phase; latency-related proteins include ORF-K12 (kaposin), -71 (viral FLICE inhibitory proteins, vFLIP), -72 (v-cyclin), -73 (latency-associated nuclear antigen, LANA), and -10.5 (vIRF3), as well as several other factors, such as microRNAs (miRNAs). When various cell types are infected with KSHV, a latent phase usually ensues in all cases following the infection. The length of the latency is also determined by the type of infected cells before the replication phase takes place [104]. KSHV infection can switch from the latent to the lytic replication phase, as the virus infects and affects various cell types, including monocytes, endothelial cells, and B cells, among others [105,106]. The switch is mediated by a replication and transcription activator (RTA) and encoded by ORF-50 [107]. The actions of certain signaling mediators, including those emanating from hypoxia, oxidative stress, cytokines, and specific chemicals, are necessary to activate RTA [106]. After the switch, the lytic replication phase is characterized by gene products that are involved in cell damage, progeny virion production, and their release. Besides the proliferation of virions, KSHV-related proteins can inhibit the cell cycle, arrest cell growth, innate defense, and apoptosis in infected cells [105,106,108]. Some expressed KSHV genes are capable of inducing angiogenesis and inflammatory responses, leading to the survival of infected cells and the development of KSHV-related illnesses [109,110]. The downregulation of major histocompatibility complex (MHC) proteins, with adaptive immune functions, is mediated by interferon response factors (vIRFs) and other factors (K-3 and K-5), leading to ineffective T cells and immune evasion [111,112]. Particularly, vFLIPs are encoded by KSHV proteins that affect NF-kB and contribute to the pathogenesis of KSHV-associated diseases [113,114]. Effector proteins, such as viral Interleukin-6 (vIL-6), can activate the JAK/STAT pathway and subsequently increase VEGF and angiogenic activities, as well as symptomatic signs of certain illnesses [115,116]. Several genes upregulate the expression and functions of IL-6 proteins, such as those encoded by kaposin B and ORF-4 [117]. LANA is another protein that has important functions and could inhibit the actions of p53 and pro-apoptotic proteins in infected cells [118].

**Figure 3:**
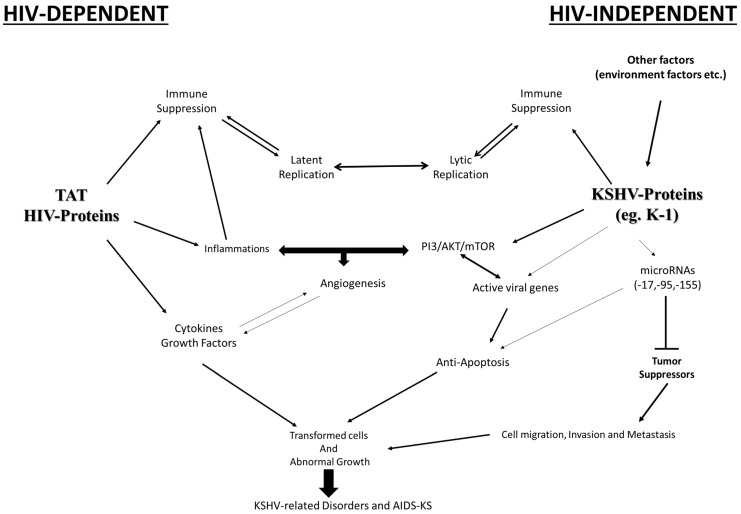
Implications of TAT HIV and KSHV proteins in mediating KSHV-related disorders in the presence and absence of HIV infection. Viral proteins can suppress immune responses, leading to lytic replication. KSHV infection induces effectors that can alter the signaling pathway PI3/AKT/mTOR, which in turn mediates inflammatory reactions, angiogenesis, and further anti-apoptotic effects in transformed or abnormal cells. Inflammations can also be triggered by TAT or other HIV proteins that promote the release of cytokines and growth factors, thereby mediating viral proliferation and cell damage. KSHV-related diseases are induced more readily in the presence of KSHV-HIV co-infection.

The probability of developing KSHV-related diseases is greater in patients co-infected with HIV than in the general population [98,118]. Chronic inflammatory responses associated with HIV status have been shown to induce KSHV-related diseases [3,54,119]. Equally, patients with HIV have a higher risk of developing other pathologies than other population subgroups [120,121]. Most viral prevalence rates are almost 3-fold and higher in those with HIV than rates reported in uninfected individuals in West Africa [48,57,67,83,98,101,119,122]. Approximately 70% of the global HIV-related burden is found in SSA, and, unfortunately, very little effort has been made to understand the HIV and KSHV co-infection in SSA, the most affected region of the world [48,123]. According to studies conducted in various parts of SSA, KSHV induces pathologies more readily when in co-infection with HIV-1 than HIV-2 [35,48,53,124,125]. The main role of HIV-1 is to establish latency in CD4 memory cells, which are typically found at rest in monocytes and macrophages [53,126]. CD4 cells play an essential role in controlling KSHV infection by maintaining the virus in a latent phase for extended periods [124,127]. The induced and gradual shutdown of immune functions through the loss of CD4 cell activity is a major setback for the switch and activation of latent replication and the lytic phase [124,126,127]. The activation is accompanied by the production and release of cytokines and other mediators that further activate KSHV genomics [128]. The effects of KSHV genes, immune suppression, inflammatory reactions, viral microRNAs, and other surrounding factors may work together to promote the development of related diseases. The HIV-encoded TAT protein is one of the key activators of these released mediators, creating a favorable microenvironment for the emergence of pathologies [124,129,130]. The TAT protein can penetrate and affect a wide range of cells, creating immune disruption, co-infections, and opportunistic diseases [131]. Clearly, HIV infection is a major factor and influence on KSHV infectiousness, angiogenesis, and signaling pathways. An immune dysfunction or the host’s deteriorated immunity is an essential cofactor in the development of related disorders, including KS tumorigenesis. Figure 3 summarizes the principal roles of TAT and KSHV proteins in mediating KSHV-related disorders and others in the presence and absence of HIV infection.

## 3. Kaposi’s Sarcoma Malignancy

### 3.1. Epidemiology and Carcinogenesis

Kaposi’s sarcoma (KS) is an angio-proliferative tumor that primarily affects the skin after a multifactorial pathogenesis mediated by immune dysfunction. The spindle cells that form the tumor are derived from endothelial and infected immune cells with KSHV [132]. The tumor was first described by Moritz Kaposi as unusual, pigmented lesions on the skin of elderly European and Mediterranean men [133]. It is now known as the classic type of KS and remains prevalent in those regions. Another type, known as the iatrogenic or immune suppressive type, was identified in the late 1960s among patients who had undergone organ transplantation and such procedures [134,135]. The endemic type of KS is characterized by lymphadenopathy, first described in 1914, and commonly observed in children as young as 3 years old to individuals in their 40s and 50s in SSA [72,135]. With the emergence of HIV from the early 1980s, a new type of epidemic KS was described among young MSM in the United States, which is known as AIDS- or HIV-related KS [135,136]. Recently, a fifth type was described in young and middle-aged MSM, who were uninfected or without HIV infection [49,137]. In comparison with the general population, higher incidences of KS have been reported: 200-fold higher in solid organ transplant recipients and 500-fold higher in AIDS-related patients. The most aggressive forms of the tumor have been observed in the presence of HIV-related types, in which the tumor can be found in the mucosae or visceral organs [49,135,138,139].

The incidence of HIV-related KS may be considerably reduced after implementing combination antiretroviral therapy (cART) in HIV patients [140]. Thus, with proper management of HIV infection, the incidence of KS can be controlled, especially the HIV-related type that represents around 70% of the global burden of KS [135,140,141]. Prior to the HIV epidemic, KS was extremely rare in females, as it predominantly affected men according to African and Ugandan-based records [59]. However, KS has become as common in women as in men across the continent, even in areas where KS was unknown, but KSHV had been prevalent [59,142]. Henceforward, HIV infection facilitates both KSHV infection and virulence, as well as subsequent KS carcinogenesis. The presence of KSHV infection is necessary but not sufficient to induce KS; when considered alone, KSHV infection has been reported in most cases of KS, which usually develop in the context of immune deficiency or in the presence of an inducer of immune suppression [49,135,142]. After being infected with KSHV, most people who develop KS usually have a weakened immune system or genetic vulnerability. Certain countries, including Gambia and the Ivory Coast, have a high prevalence of KSHV infection, but a relatively low incidence of KS [48,59,142]. An anti-tumor or tumor-suppressor protein, well-known as the p53 protein, plays crucial regulatory roles (cell cycle arrest, DNA repair, senescence, and apoptosis) in response to various stimuli, including stress, redox signals, genotoxic agents, hypoxia, and oncogenic activation. In oncogenesis, p53 is most frequently mutated in a variety of human cancers [143]. The viral genes of KSHV interfere with p53 at several levels, altering its functions [144]. Viruses may work together, in so many ways, with co-carcinogens to act either as initiators or as promoters, or even both, all depending on their prevalent effects: mutagenic (herpesviruses) or epigenetic (papillomaviruses) [142]. Other cofactors are required to effectively progress to the development of chronic infections or KS tumors. The diversity of clinical outcomes indicates multiple factors (immunologic, genetic, and environmental) are necessary for oncogenicity [142,145].

### 3.2. Other KS-Facilitating Factors and Mitigating Measures in SSA

After analyzing the geographical distribution of KS and KSHV-HIV co-infection and considering the high prevalence of KSHV, as well as the incidence of KSHV-related diseases in SSA, it became clear that additional factors play essential roles in the onset of the associated diseases. Pregnant women and children under the age of 5 are considered among the most vulnerable and affected subgroups by KSHV-infection in SSA. Similarly, these subgroups are also vulnerable to malaria, which is a parasitic disease that affects about one-fifth of the SSA population [146,147,148]. Approximately 233 million new cases were reported in 2022 worldwide, with the top 20 malaria-affected countries being located in Africa. Four SSA countries (Nigeria, DR Congo, Tanzania, and Mozambique) accounted for almost half of global cases [146,147,148,149]. Malaria dramatically affects the population of the SSA region, not only by killing the citizens but also by leaving long-term health problems in the survivors. Recently, reports have shown that DNA damage caused by the malaria parasite increases the risk of converting infected cells into cancerous ones [150]. Given the occurrence of KSHV infection, a patient with a malarial history and an increased risk of cell conversion is more likely to develop KS than one without malaria, cell convertibility, or any related health-threatening issue. Furthermore, evidence has shown that patients infected with either Epstein–Barr virus, hepatitis C virus, HIV, helicobacter pylori, or plasmodium falciparum are all at higher risk of developing B-cell lymphoma [151]. Also, endemic Burkitt lymphoma is one of the most commonly diagnosed cancers in children, occurring at higher incidences in areas where Plasmodium falciparum (malaria parasite)-induced infection is endemic [152].

Having several concurrent infections would weaken immune functions, facilitating cell convertibility and pathogenesis. Thus, having KSHV infection, plus malaria or any other infection commonly associated with regions of SSA, would increase the risk of developing KS. Additionally, several drugs, such as antimalarial and quinine derivatives, can act as immunosuppressive agents, interfering with immune responses during infections or immunization [150,151,152,153]. The synergetic effects of certain active/therapeutic agents could facilitate the onset of AIDS-related KS in patients with HIV [154,155]. Certain effects of antimalarial agents on KS induction, for example, seem controversial and can either favor or inhibit KS pathogenesis. Thus, among the elements to be considered when attempting to address the high KSHV prevalence and KS incidence in SSA, the medical history of patients should be thoroughly assessed prior to treatment.

In mitigating KSHV transmission, certain behavioral habits, such as food mastication and sharing food and drinks, should be avoided, as they have been linked to KSHV positivity in African children [156,157]. In South Korea, in individuals without HIV, smoking and alcohol consumption, as well as metabolic disorders, have been associated with a risk of developing KS [158]. In another study conducted in the USA, KSHV positivity and KS pathogenicity were linked to alcohol consumption in women, men, and MSM studied subgroups [159]. Some citizens from SSA countries with KSHV endemicity are among the heaviest alcohol consumers, occupying top rankings on the continent, such as Tanzania, Burkina Faso, South Africa, Uganda, and Gabon [160]. In South Africa, smoking and alcohol consumption were 5 and 3 times higher in males than in females. Positive associations between KS pathogenicity and heavy alcohol consumption (vs. non-drinking) and heavy smoking (vs. never-smoking) were established [161]. The link between smoking and the mechanisms that cause certain diseases related to the respiratory tract is well documented. Drinking alcohol has been directly linked to an elevated risk of various solid cancers. Alcohol and its metabolites can readily damage DNA (disrupt synthesis, repair, and methylation), causing inflammation, oxidative stress, and lipid peroxidation, all eventually leading to cancer formation [162]. The development of KS may certainly depend on various modulators, such as medical history, genetic disposition, alcohol or drug intake, lifestyle, healthcare organization, and sanitary and environmental conditions. The diurnal climatic conditions and year-round high temperatures of certain SSA regions facilitate the proliferation of infectious agents and diseases [162,163]. Expectedly, Africa bears the largest burden of KS worldwide, accounting for 73% of all incident cases and approximately 87% of all deaths reported in 2020. These statistics remain high due to limited access to adequate healthcare facilities [164].

## 4. A Potential “All-in-One” Solution

Most KSHV infections are asymptomatic till later stages, rendering most therapeutic efforts less effective, if not ineffective. All existing treatment modalities aim to alleviate the burden or slow the progression of associated diseases, including KS tumorigenesis. For example, highly active antiretroviral therapy (HAART) could also be used to prevent the appearance of lesions in AIDS-related KS patients [51,165]. Therapeutic innovations are encouraged, and combination therapies are preferred, becoming increasingly popular. The artemisinin-based combination treatments (ACTs) that integrate artemisinin derivatives (ART and ARM) and photochemotherapeutic agents (photosensitizers, PSs), like 5-Aminolevulinic acid (Ala), can generate toxic oxygen radicals upon illumination to destroy intraerythrocytic and blood parasites [166]. Such combination approaches could also be used to treat hematologic cancers. Efforts to search for novel therapeutic approaches for cancer and malaria led to the development of several hybrid compounds. Some hybrid molecules containing artemisinin have displayed significant anticancer, antimalarial, or antiviral activity in vitro and in vivo, with the potential to overcome drug resistance, minimize toxic side effects, and achieve effective targeting [165,167]. Artemisinin derivatives have demonstrated anticancer effects in resistant cell lines, exhibiting anticancer activities against cancer cells in various organs, including the liver, stomach, skin, cervix, head, neck, breast, lung, pancreas, and glioma [168,169,170].

Photodynamic therapy (PDT) is an alternative treatment used to treat certain skin conditions and select types of cancer, including those of the neck and head. This therapy is almost abstruse in Africa and involves photodynamic actions when photosensitizing molecules (PSs) absorb light energies (photons) and dissipate absorbed photons by transferring them to biological acceptors (cellular oxygen in most cases), generating reactive oxygen species (ROS), which cause cell damage and death [171,172]. Many etiological agents associated with tropical diseases are susceptible to photodynamic actions. Several reports have indicated that PDT is effective in treating many tumors, as well as inactivating the replication of fungi, bacteria, and viruses, thus known as photodynamic inactivation (PDI) [173,174].

An amphiphilic-mediated PDI inhibited herpes simplex virus-1 (HSV-1) without creating obvious cytotoxicity in the host microenvironment. The viral replication of HSV-1 was effectively inhibited, and this finding could be used as a preventive measure [175]. The curcumin-mediated PDI efficacy in treating infected cells has been reported both in vitro and in vivo [176]. The efficacy of PDI against infectious agents, both in vitro and in vivo, was reported and found not necessarily dependent on the PS used [177]. The combined actions of Acyclovir, an antiviral (herpes) agent, and 5-Ala (PS)-mediated PDT led to better results, including edema and tingling reduction from day 1 of the treatment when compared to Acyclovir mono-based treatment [178]. PDT promoted cell damage in cancerous and infected cells, effectively addressing the recurrent issue of antimicrobial resistance associated with other treatment modalities [179,180]. One of the main disadvantages of PDT is its limited effectiveness in treating solid tumors or diseased cells in deeper tissues, where concerns include limited light penetration and oxygen supply (hypoxia). The most outstanding results of PDT are seen with superficial conditions, such as those induced by KS malignancy; thus, there is no doubt that PDT is suitable for KS lesions and KSHV-related conditions [181].

The PI3/AkT/mTOR signaling pathway is critical in developing all the conditions as described above. An effective treatment should be able to impair this signaling pathway. Recently, Hematoporphyrin (HpD, a well-known PS)-mediated PDT was proved to be effective in inducing apoptosis and suppressing the migration of human esophageal squamous cell carcinoma by regulating the PI3/AkT/mTOR signaling pathway [182]. It is important to note that not only was the signaling pathway inhibited, but also cellular apoptosis and autophagy were induced; these are essential programmed mechanisms and responses for efficient cancer treatment when HpD-mediated PDT is combined with a PI3K inhibitor. The therapeutic outcomes were enhanced when compared to those achieved with HpD-mediated PDT alone, which had already yielded good therapeutic outcomes [182]. Another recent study reported that Ala-mediated PDT in the presence of gold nanotriangles impaired cell survival and the PI3K/AkT signaling pathway, causing mitochondrial-dependent death and achieving excellent targeting of the breast cancer cells with Triphenylphosphonium [183]. The PI3K/AKT/mTOR signaling pathway plays a significant role in various human cancers, which can pave the way for the development of anticancer therapeutics. PDT can be a potential option for managing various conditions that involve this signaling pathway.

## 5. Conclusions

Besides the endemic aspects of infections, other factors seem to be determinant in developing KSHV-induced conditions in any given specific subgroup. While some conditions do not affect the course of the viral infection, others exacerbate its effects and contribute to severe scenarios of subsequent diseases. The KSHV infectious agent disturbs inflammatory and immune responses in individuals infected with HIV. KSHV infection alone is not sufficient to cause KSHV-associated pathologies. It differs according to the specific features of the population of interest, medical status, and history, as well as KSHV genotypes, among other factors. HIV infection and subsequent effects have tremendous impacts on KSHV infectiousness and angiogenesis, and bring about turbulence in signaling pathways. Furthermore, the course of infection or status of patients may well be exacerbated by several dynamics, including regional endemicity for certain conditions (HIV and/or malaria, etc.), lifestyle, and healthcare facilities, which could explain KS development and incidence in most SSA inhabitants. Combined treatment options should be introduced to better manage and mitigate the complexity and burden of KS in SSA. Alternative approaches, such as photodynamic therapy, a multi-targeted and well-suited option for KS, present numerous advantages and practical solutions for SSA.

## Figures and Tables

**Table 1:** Summary of the estimated KSHV prevalence and KS incidence rates in certain SSA countries.

SSA Regions	Countries	KSHV-Subtypes	Seroprevalence	KS-Incidence (Per 1000)	Ref.
Central	Cameroon	A5, B, P	>50%	4–8	[25,72,75,76,77,78,79,83,84]
	Gabon	A5	25–50%	2–4	[33,59,72,76,80]
	Congo Brazzaville	B, M	25–50%	0–0.5	[85,86]
Southern	South Africa	A5, B, N	>50%	4–8	[59,64,72,73,74,87]
	Zambia	A5, B, C, Z, M, N, P	>50%	1–8	[59,72,75,88]
	Zimbabwe	A5	>50%	4–8	[8,58,59,71,72]
Eastern	Uganda	A5, C, F, P, M	>50%	1–8	[58,59,60,62,64,68,72,77]
	Tanzania	A5, B, P	>50%	2–4	[59,65,66,72,89]
	Kenya	A5, B, C, F, P	>50%	0.5–2	[69,70,72,90]
Western	Ghana	A5, B	>50%	0–0.5	[58,59,72,91,92]
	Nigeria	A5	>50%	0.5–2	[57,59,65,72,74]
	Burkina Faso	A5	>25%	0–0,5	[56,59,93,94]

SSA = Sub-Saharan Africa; KSHV = Kaposi’s sarcoma-associated herpes virus; KS = Kaposi’s sarcoma.
